# Polymorphisms and minihaplotypes in the *VvNAC26* gene associate with berry size variation in grapevine

**DOI:** 10.1186/s12870-015-0622-2

**Published:** 2015-10-23

**Authors:** Javier Tello, Rafael Torres-Pérez, Jérôme Grimplet, Pablo Carbonell-Bejerano, José Miguel Martínez-Zapater, Javier Ibáñez

**Affiliations:** Instituto de Ciencias de la Vid y del Vino (CSIC, Universidad de La Rioja, Gobierno de La Rioja), Carretera LO-20 salida 13, Finca La Grajera, 26007 Logroño, Spain

**Keywords:** *Vitis vinifera* L, Association genetics, Fruit growth, Fruit size, Haplotype, NAC transcription factor, Phylogenetics

## Abstract

**Background:**

Domestication and selection of *Vitis vinifera* L. for table and wine grapes has led to a large level of berry size diversity in current grapevine cultivars. Identifying the genetic basis for this natural variation is paramount both for breeding programs and for elucidating which genes contributed to crop evolution during domestication and selection processes. The gene *VvNAC26*, which encodes a NAC domain-containing transcription factor, has been related to the early development of grapevine flowers and berries. It was selected as candidate gene for an association study to elucidate its possible participation in the natural variation of reproductive traits in cultivated grapevine.

**Methods:**

A grapevine collection of 114 varieties was characterized during three consecutive seasons for different berry and bunch traits. The promoter and coding regions of VvNAC26 gene (VIT_01s0026g02710) were sequenced in all the varieties of the collection, and the existing polymorphisms (SNP and INDEL) were detected. The corresponding haplotypes were inferred and used for a phylogenetic analysis. The possible associations between genotypic and phenotypic data were analyzed independently for each season data, using different models and significance thresholds.

**Results:**

A total of 30 non-rare polymorphisms were detected in the VvNAC26 sequence, and 26 different haplotypes were inferred. Phylogenetic analysis revealed their clustering in two major haplogroups with marked phenotypic differences in berry size between varieties harboring haplogroup-specific alleles. After correcting the statistical models for the effect of the population genetic stratification, we found a set of polymorphisms associated with berry size explaining between 8.4 and 21.7 % (R^2^) of trait variance, including those generating the differentiation between both haplogroups. Haplotypes built from only three polymorphisms (minihaplotypes) were also associated with this trait (R^2^: 17.5 – 26.6 %), supporting the involvement of this gene in the natural variation for berry size.

**Conclusions:**

Our results suggest the participation of *VvNAC26* in the determination of the grape berry final size. Different *VvNAC26* polymorphisms and their combination showed to be associated with different features of the fruit. The phylogenetic relationships between the *VvNAC26* haplotypes and the association results indicate that this nucleotide variation may have contributed to the differentiation between table and wine grapes.

**Electronic supplementary material:**

The online version of this article (doi:10.1186/s12870-015-0622-2) contains supplementary material, which is available to authorized users.

## Background

Grapes are one of the most valuable and extensively cultivated fruits, mainly grown for their transformation into wine, juice or raisins, and for direct consumption as fresh fruit [1]. The cultivated grapevine (*Vitis vinifera* subsp. *sativa*) derives from its wild ancestor (*Vitis vinifera* subsp. *sylvestris*) through several domestication processes [2, 3]. Archeological findings suggest that primary domestication events could have taken place between the seventh and fourth millennia BC in the Near East region located between the Black and Caspian seas [4–6]. From there, those initial cultivars would had been spread by human civilizations in different directions [4]. Additional secondary domestication events and spontaneous hybridizations among selected individuals and local wild populations likely contributed to the evolution of current cultivars, since the ancestor species was present all around the Mediterranean sea [7, 8]. Current cultivated grapevine shows important modifications compared to its wild relative, including the radical change in the sexual form of the plant - from dioecy to hermaphroditism-, and the increase in the number of berries per bunch and their individual size [4, 5, 9–11].

As for other crops, fruit size is a trait that was preferentially selected during the domestication of grapevine [4, 10–12]. Because of the selection to increase yield, berries from cultivated varieties are larger than those from their wild ancestor [2, 4]. Moreover, specific berry features have been selected for either wine or table grape production [1, 4]. In this light, cultivars with large and fleshy berries are preferred for their use as table grape varieties, whereas cultivars with smaller and juicier berries and a higher skin-to-flesh ratio are preferred for winemaking [2, 13]. The existence of divergent selection has likely contributed to the large diversity that can be found nowadays for berry morphology [11, 14]. Variation in berry and bunch traits allowed the distinction of three morphotype groups (or *proles*): the *occidentalis*, grouping the small-berried wine cultivars of Western Europe, the *orientalis*, composed by the large-berried table cultivars of Central Asia, and the *pontica*, with cultivars with an intermediate phenotype and grown around the Black Sea and in Eastern Europe [15]. Relationships between these morphotypes and different nuclear and chloroplast haplotypes have been proposed [7, 16], suggesting the use of different genetic pools for the development of wine and table cultivars in different geographical regions. Recently, Bacilieri et al. [2] studied the genetic structure of more than 2000 grapevine accessions, identifying the existence of three main genetic groups in agreement with the morphotypes classification. Additional stratification identified five different genetic groups: a group of wine and table cultivars from the Iberian Peninsula and Maghreb (S-5.1), a group of table cultivars from Far- and Middle-East countries (S-5.2), a group of wine cultivars from West and Central Europe (S-5.3), a group comprising mostly bred table grape cultivars from Italy and Central Europe (S-5.4), and a group of wine cultivars from the Balkans and East Europe (S-5.5) [2]. In a similar approach, Emanuelli et al. [3] identified four genetic groups in 1659 sativa grapevine genotypes by means of a set of SSR markers: a group of Italian/Balkan wine cultivars (VV1), a group of Mediterranean table/wine grapes (VV2), a third group with the Muscats varieties (VV3), and a group of Central European wine grapes (VV4).

To date, several quantitative trait loci (QTL) for berry size have been detected through the analysis of different grapevine progenies from crosses involving either wine or table varieties as parents [17–22]. Although this approach has provided useful information for the analysis of the trait, the results are usually restricted to the analyzed progenies [23]. In this sense, association mapping searches for variation in a much broader genetic context, enabling the exploitation of the diversity that is naturally present in a crop as a result of centuries of evolution [24]. Two types of association methods are currently used for the dissection of complex traits: genome-wide association studies (GWAS) and candidate-gene association mapping [24, 25]. The last one is a hypothesis-driven approach that requires of a candidate gene selected on the basis of previous results obtained from genetic, functional or physiological studies [24, 25]. This approach has been successfully applied in grapevine studies providing evidence for the role of *VvMyb* genes in the anthocyanin content of berry skin [26, 27], *VvDXS* in Muscat flavour [28], *VvPel* and *VvGaI1* in berry texture [29, 30], *VvAGL11* in seedlessness [31], and *VvTFL1A* in flowering time, berry weight and bunch width [32].

NAC domain-containing proteins [from Petunia *NO APICAL MERISTEM* (*NAM*) and Arabidopsis *TRANSCRIPTION ACTIVATION FACTOR* (*ATAF1,2*) and *CUP-SHAPED COTYLEDON* (*CUC*)] are one of the largest families of plant-specific transcription factors, being characterized in a wide range of land plants [33]. NAC proteins contain a highly conserved domain at the N terminus (NAC domain) and a highly divergent transcriptional regulatory region in the C-terminal region that determine the specific function of the protein [33, 34]. The NAC domain consists of approximately 150-160 amino acids, and is divided into five well-conserved subdomains [34]. This region holds DNA binding activity and can be responsible for protein binding and dimerization [34, 35]. This transcriptional factor family has been related to different developmental and morphogenetic processes in Arabidopsis [36–41] and other species [42–47].

Regarding grapevine, 74 different *NAC-like* genes (*VvNAC*) have been identified in the reference genome version 0 [48] and 75 in version 1 [49]. According to their homology to *AtNAC* genes, some have been predicted to play different roles during grapevine development [48]. In a recent phylogenetic analysis performed between the NAC sequences from *V. vinifera*, *Arabidopsis thaliana*, *Oryza sativa* and *Musa acuminata*, VvNAC26 showed to be the closest homologue to *Arabidopsis* NAC-LIKE, ACTIVATED BY AP3/PI (NAP, also known as AtNAP or ANAC029) [50]. *AtNAP* is a target gene of the flower homeotic transcription factors *APETALA3*/*PISTILLATA* (*AP3*/*PI*) [38, 51], two MADS-box genes required for the determination of petal and stamen identities during flower development in Arabidopsis. In grapevine, Fernandez et al. [52] identified the specific over-expression of a putative *AtNAP* homolog during the development of flowers and berries of the extreme fleshless berry *flb* mutant of the cultivar Ugni Blanc, suggesting the involvement of this NAC transcription factor in berry flesh morphogenesis. In fact, *VvNAP* is also up-regulated in berries of cvs. Ugni Blanc and Cabernet Sauvignon before the onset of ripening [52], suggesting its involvement in normal berry development.

Considering the function of *NAP* in Arabidopsis cell growth [38] and the likely involvement of its grapevine homolog in berry development and growth [52], *VvNAC26* was selected as a candidate gene to analyze its contribution to fruit size natural variation in the cultivated grapevine. *VvNAC26* was sequenced in a set of table and wine grapevine varieties that were described over three consecutive years for nine berry and bunch traits. Additional tests to evaluate the linkage disequilibrium (LD) between the polymorphisms detected along the *VvNAC26* sequence and the likely stratification of the grapevine varieties used in this work were performed to reduce the presence of false positive marker/trait associations. Moreover, *VvNAC26* haplotypes inference and analyses gave us insights of the likely evolution of the gene considering the origin of the varieties used in this study. Lastly, reduced ancestral haplotypes (minihaplotypes) showing association with berry size were identified.

## Methods

### Plant material

A total of 114 grapevine varieties (including 111 *V. vinifera* cultivars and three inter-specific hybrids) held at the Grapevine Germplasm Collection of the Instituto de Ciencias de la Vid y del Vino (ICVV,FAO Institute Code: ESP-217) were considered (Additional file 1). Most of the cultivars used in this work come from Spain, France, Portugal and Italy. They are maintained under the same agronomical conditions in two separated experimental plots: “*Finca Valdegón*” (Agoncillo, La Rioja, Spain) and “*Finca La Grajera*” (Logroño, La Rioja, Spain). Plants at “*Finca La Grajera*” (5 years old) come from scions taken from “*Finca Valdegón*” (20-30 years old). This set of varieties was described in three consecutive vintages: 2011 and 2012 (in “*Finca Valdegón*”) and 2013 (in “*Finca La Grajera*”). Information on the origin, main use and pedigree of the varieties was obtained from the *Vitis* International Variety Catalogue (VIVC, http://www.vivc.de, accessed: March 2015) (Additional file 1).

### Phenotypic data

Due to inter-annual fluctuations, all grapevine varieties could not be described for the three seasons. Thus, 98, 104 and 97 varieties were sampled in 2011, 2012 and 2013 respectively. As a rule, ten mature bunches (at growth stage E-L 38 [53]) were collected per variety and characterized for nine berry and bunch traits (Table 1) as described previously [54, 55]. To better fit the assumption of normality in the statistical analyses, the variable “Bunch weight” was square-root transformed, whereas variables “Berry weight” and “Berry volume” were logarithmically transformed. Phenotypic distribution of the traits considered in this study can be found in Additional file 2. Correlations between traits and seasons were performed with SPSS v.22.0 (IBM, Chicago, IL, USA) using the Pearson correlation coefficient.

**Table 1:** Bunch and berry traits analyzed in this study

	2011			2012			2013		
	Mean ± s.d.	Min.	Max.	Mean ± s.d.	Min.	Max.	Mean ± s.d.	Min.	Max.
Berries per bunch	136.3 ± 47.5	42.5	272.0	108.9 ± 39.0	37.8	210.2	123.4 ± 50.4	42.7	285.9
Berry length (mm)	14.0 ± 3.0	9.9	23.4	13.1 ± 2.6	8.9	23.8	16.3 ± 3.6	10.6	28.0
Berry volume (mL)	1.5 ± 0.8	0.6	5.0	1.2 ± 0.6	0.4	3.3	2.1 ± 1.2	0.6	7.2
Berry weight (g)	1.6 ± 0.8	0.6	5.4	1.3 ± 0.6	0.5	3.4	2.2 ± 1.2	0.6	7.5
Berry width (mm)	13.2 ± 2.0	9.5	19.1	12.7 ± 1.9	9.3	18.6	14.9 ± 2.5	10.4	24.0
Bunch length (cm)	16.8 ± 3.7	10.3	27.7	14.6 ± 3.5	7.6	25.1	18.2 ± 4.7	7.5	30.5
Bunch weight (g)	227.9 ± 114.8	69.9	589.4	145.9 ± 74.0	48.7	392.2	285.1 ± 151.6	56.0	726.9
Bunch width (cm)	10.8 ± 2.2	6.4	15.7	8.9 ± 1.8	5.6	15.3	11.6 ± 2.9	5.8	18.3
Seeds per berry	2.0 ± 0.5	0.0	3.2	2.2 ± 0.6	0.0	3.8	1.9 ± 0.5	0.0	3.5

Mean, standard deviation (s.d.), minimum (Min.) and maximum (Max.) values obtained in 2011 (*n* = 98), 2012 (*n* = 104) and 2013 (*n* = 97)

### Genotypic data

Young leaves from the 114 grapevine varieties were sampled and stored at -80 °C until DNA extraction. Genomic DNA was isolated using the DNeasy Plant Mini kit (Qiagen, Valencia, CA, USA), following the instructions provided by the manufacturer. DNA was qualitatively and quantitatively evaluated by visual comparison with lambda DNA on ethidium bromide-stained agarose gels (0.8 %), and a NanoDrop 2000 spectrophotometer (Thermo Scientific, Wilmington, DE, USA). Nine nuclear SSR loci (VVS2, VVMD5, VVMD27, VVMD28, ssrVrZAG29, ssrVrZAG62, ssrVrZAG67, ssrVrZAG83 and ssrVrZAG112 [56]) and four chloroplast SSR loci (cpSSR3, cpSSR5, cpSSR10 [57] and cpSSR9 [58]) were analyzed in the 114 varieties. Polymerase chain reaction (PCR), separation of fragments, and data analysis were performed following the procedure detailed in Ibáñez et al. [59]. Pair-wise multilocus comparison with the ICVV nuclear and chloroplast SSR database and The European *Vitis* database (http://www.eu-vitis.de) was performed for the genetic identification of the variety. Chlorotypes were named according to Arroyo-García et al. [7].

The *VvNAC26* gene (VIT_01s0026g02710), including 1000 bp in the promoter region according to grapevine 12X V1 gene predictions (http://genomes.cribi.unipd.it/gb2/gbrowse/public/vitis_vinifera/), was sequenced together with other set of genes (data not shown). A region of 2184 bp (chr01_12442003:12444186) was targeted for next-generation sequencing (NGS) following a protocol based on the Agilent SureSelect Target Enrichment workflow (http://www.genomics.agilent.com). Paired-end libraries with an insert size of approximately 350 bp were sequenced in an Illumina HiSeq 2000 platform by BGI company (http://www.genomics.cn/en). Target enrichment and sequencing were carried out by BGI. Resulting reads had an average size of 90 nt, and were aligned to the whole 12X V1 *Vitis vinifera* PN40024 reference genome [60] with Bowtie 2 [61] using the following command line settings: --phred64 --end-to-end -N 0 -L 25 --gbar 2 --np 6 --rdg 6,4 -X 400 --fr –no-unal. The variant caller utility implemented in the SAMtools package [62] was used to detect polymorphisms (SNPs and INDELs) between the reference genome and each of the 114 sequenced varieties. These initially detected polymorphisms were filtered to generate a consensus genotype per variety by means of an ad hoc Perl script in which thresholds of quality score, read depth and frequency of base calls were considered (the source code of the script and a complete description of filtering parameters are available at https://github.com/ratope/VcfFilter). To verify the consistency of variant calling, polymorphisms were individually checked with the Integrative Genomics Viewer (IGV) software [63]. Polymorphisms are named as suggested by Fernandez et al. [32], using the abbreviation “IND” for the designation of INDELs. Linkage disequilibrium (LD) was estimated considering polymorphisms with a minor allele frequency (MAF) higher than 5 %, by calculating the genotypic correlation coefficient (*r*^2^) together with its associated *P*-value by a built-in function of TASSEL v.3.0 (http://www.maizegenetics.net/) [64], and LD-blocks were determined considering a critical *r*^2^ value of 0.8.

Prediction of the likely effect of the detected polymorphisms in the encoded protein was carried out with SnpEff v.4.0 [65], and effects of single amino acid substitutions on protein function were predicted in parallel with SNAP [66] and PROVEAN [67] utilities. We also checked for their likely effect on the mRNA secondary structure using two independent web-based applications: RNAsnp [68] and RNAstructure [69].

To predict the likely effect of the polymorphisms located in the promoter, we carried out the detection of the putative regulatory motifs with PlantCARE [70].

### *VvNAC26* haplotypes and nucleotide diversity analyses

Haplotype inference and diplotype (haplotype pair) estimation were performed with the partition-ligation-expectation-maximization (PLEM) algorithm [71] implemented in PHASE v.2.1, using default settings [72]. Haplotype clustering was carried out by SPSS v.22.0 (IBM, Chicago, IL) using Ward’s hierarchical method. Haplotypes were tested for recombination using the MaxChi, Chimaera and 3Seq algorithms implemented in the Recombination Detection Program v.4.46 (RDP4) [73] with default settings. A median-joining network [74] was constructed for the inferred haplotypes with the software Network v.4.6 (www.fluxus-engineering.com). Molecular diversity was evaluated through the calculation of the nucleotide diversity (π) [75] and the Watterson θ estimate [76] with DnaSP v.5.10 [77]. This software was also employed to obtain insights for testing likely deviations from neutrality, through the computation of Tajima’s *D* [78] and Fu and Li’s D* [79] tests. They were calculated for the whole set of haplotypes and separately for the genetic groups detected by STRUCTURE v.2.3, as suggested in Fernandez et al. [32].

### Population genetic structure and kinship matrix

The number of genetic groups in the grapevine collection analyzed was estimated by the Bayesian approach implemented in the software package STRUCTURE v.2.3 [80]. It was run on the basis of the nine nuclear SSR markers using an admixture model with uncorrelated allele frequencies. This model was tested in a number of hypothetical genetic groups ranging from 1 to 15, with 100,000 burn-in iterations followed by 150,000 Markov Chain Monte Carlo (MCMC) iterations for an accurate estimation. Each number of likely genetic groups was performed in 5 independent runs to verify the consistency of the results. The most probable number of genetic groups was assessed following the criteria proposed by Evanno et al. [81], as implemented in STRUCTURE HARVESTER [82]. Once the optimal number of genetic groups was detected, we used CLUMPP v.1.1 [83] to align the 5 different runs, and the consensus matrix (*Q*) was used for association analyses. DISTRUCT v.1.1 [84] was used for the graphical visualization and analysis of the population structure. Grapevine varieties were assigned to a genetic group when its membership coefficient was 0.75 or higher; genotypes with no scores over this value were considered as “admixed”. As suggested by Ruggieri et al. [85], the effect of the population structure on the variation of the traits considered was evaluated by multiple regression analysis, performed with SPSS v.22.0 (IBM, Chicago, IL, USA).

A kinship matrix (*K*) was constructed for obtaining the estimators of pairwise relatedness proposed by Wang [86] for our set of varieties, using the *related* package [87] for R v.3.2.2 (http://www.r-project.org/). They were estimated on the basis of 25 SSR: the mentioned set of 9 SSR markers plus 16 additional SSR markers obtained for 102 varieties from available data previously published by Lacombe et al. [88] and de Andrés et al. [89].

### Association analyses

Association analyses between genotypic and phenotypic data were performed separately for 2011, 2012 and 2013 seasons, considering only those polymorphic sites with a MAF ≥ 5 % and the average value obtained for the bunches analyzed of each accession. Four different models were tested using TASSEL v.3.0 [64] to detect the most conservative one, using the P3D (Population Parameters Previously Determined) method and an optimum level of compression as estimation variables. The four methods tested were: Naïve model [a General Linear Model (GLM) without any correction for population structure]; Q model (a GLM model with fixed population structure as covariate); K model [a Mixed Linear Model (MLM) with kinship *K* as correction factor]; and Q + K model [a MLM model capable to correct for both population structure (*Q*) and kinship (*K*) effects [90]]. Association results indicated the last one as the most stringent one (Additional file 3), so only their results are shown and discussed.

To assess significance level, a multiple testing correction based on the number of tests was performed. It was determined considering the number of traits evaluated and the number of independent markers analyzed, which was determined by counting one polymorphism per LD-block plus all interblock polymorphisms [91]. Two thresholds for the *P*-value were considered: the first one (*P*-value ≤ 3. 27E^-4^) corresponds to the stringent Bonferroni corrected level for α = 0.05, the second one (*P*-value ≤ 6.53E^-3^) allows the appearance of one false positive per multiple testing [91].

As suggested by Carter et al. [92], association analyses were also performed between the phenotypic data and a set of reduced haplotypes (minihaplotypes, MH), which were inferred as previously detailed but considering only the most informative polymorphisms. Since nine traits were tested per year, associations showing a *P*-value lower than 5.55E^-3^ (the Bonferroni-corrected threshold for nine comparisons for α = 0.05) were considered as significant.

## Results

### Phenotypic data

A large phenotypic variation was found for the traits evaluated in our set of grapevine varieties (Table 1). Similar levels of variation have been described for these traits in different core collections [11, 32], supporting the actual adequateness of the plant material. Variation in fruit size parameters in different years was highly correlated (Additional file 4) what, in addition to high values of broad sense heritability for the studied traits in this set of varieties (data not shown), suggest the existence of a strong genetic component for the observed phenotypic variation in fruit growth-related traits. Interestingly, we found no significant correlation (or it was very low) between the number of seeds per berry and the different berry traits included in this study, in accordance with Houel et al. [11].

### Population genetic structure

The existence of population stratification can lead to spurious marker/trait associations given the geographical origin, local adaptation and breeding history of the plant material [24]. STRUCTURE analysis and Evanno’s *ΔK* method suggested the most likely existence of three genetic groups (*k1*, *k2* and *k3*) (Additional file 5) using 9 SSRs. This set of markers led to a more reliable structure (in base to knowledge on genetic and geographical origin and use of the cultivars) and more conservative association results (lower *P*-values and R^2^) than a set of 261 SNP markers (data not shown). Similarly, results using 9 SSRs were compared to those obtained using the set of 25 markers used for kinship estimation (see Material and Methods). Membership coefficients given by the 9 SSR and 25 SSR structures (both obtained by means of CLUMPP) showed a high level of significant correlation (r = 0.9; p < 0.001), and association results were similar (data not shown). Because of the presence of missing values in 12 individuals for 16 SSRs, and the sensitive of STRUCTURE to individuals poorly genotyped [93], the structure based on 9 SSR markers was further considered in this study as correction factor.

**Fig. 1:**
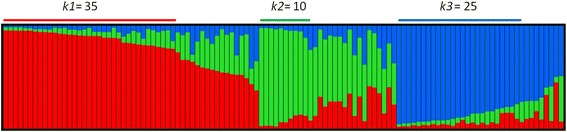
Population structure of the 114 varieties included in this study based on STRUCTURE [80]. The optimal number of genetic groups (K = 3) was set according to Evanno’s method [81]. Each variety is represented by a vertical line, divided in colored segments according to the proportion of estimated membership in the three genetic groups: *k1* (red), *k2* (green), and *k3* (blue). Considering that a variety was assigned to a genetic group if its membership is over 0.75, *k1*, *k2* and *k3* are composed by 35, 10 and 25 individuals, respectively

Considering a membership coefficient of 0.75 as a critical threshold for the assignation to a genetic group, *k1*, *k2* and *k3* include 35, 10 and 25 grapevine varieties respectively, whereas 44 varieties were considered as admixed (Fig. 1). This large proportion of admixed genotypes is in agreement with previous findings [2]. We found that this *Q* = 3 structure is consistent with both the geographic origin and the main use of the varieties considered in this work (Additional file 1). The genetic group *k1* mainly contains Iberian wine or mixed use varieties (e.g.: Airén, Palomino Fino, Tempranillo). Group *k2* is primarily composed by varieties mainly grown for producing table grapes, and typically considered part of the *orientalis* morphotype proposed by Negrul [15]. This group clusters some Muscat and Muscat-derived varieties (like Muscat Hamburg, Alphonse Lavallee and Italia), and other not related varieties (e.g.: Afus Ali, Dominga). *k3* mostly includes wine varieties from Western Europe (e.g.: Aligoté, Cabernet Sauvignon, Traminer) and some grown in the Northwest of the Iberian Peninsula (e.g.: Alfrocheiro, Alvarinho). Most of the varieties included in groups *k1* and *k3* have the morphological features of the *occidentalis* morphotype [15]. Interestingly, the structure analyses clusters Northwest Iberian wine varieties with European wine varieties, agreeing with recent results that connect those varieties through the parent-offspring relationship existing between Alfrocheiro and Traminer (or Savagnin) [94]. The three genetic groups can be identified as three of the five genetic groups proposed by Bacilieri et al. [2]. In this sense, *k1* can be related to the S-5.1 group (Wine and Table/Iberian Peninsula and Maghreb), *k2* to S-5.4 (Table/Italian and Central Europe breeds), and *k3* to S-5.3 (Wine/West and Central Europe) [2]. Moreover, they show agreement with three of the four groups suggested by Emanuelli et al. [3], with *k1* related to the VV2 group (Mediterranean table/wine grapes), *k2* to VV3 (Muscats) and *k3* to VV4 (Central European wine grapes).

Chlorotypes have been related with the geographical origin and use of the varieties, and therefore we also considered them in this work (Table 2 and Additional file 1). Chlorotype A was the most common one in the whole set of varieties analyzed (54.4 %), followed by the chlorotypes D (25.4 %) and C (14.0 %); chlorotype B (4.4 %) was only found in varieties attributed to *k2* or in admixed varieties. Chlorotype A (characteristic of Western Europe and Northern Africa [7]) was frequently found in the genetic group *k1*, whereas chlorotype C (commonly found in varieties of Central Europe [7]) was mostly found in varieties of *k3.* In this genetic group, we also found a high number of varieties with chlorotype A, due to the inclusion of Northwest Iberian varieties, as mentioned above.

**Table 2:** Distribution of chloroplast haplotypes

Chlorotype	A	B	C	D	non-vinifera
Global	62	5	16	29	2
k1	29	-	-	6	-
k2	1	3	1	5	-
k3	11	-	8	5	1
Admixed	21	2	7	13	1

Frequencies are shown for the global collection (*n* = 114 varieties) and in the three genetic groups detected by STRUCTURE: *k1* (*n* = 35), *k2* (*n* = 10) and *k3* (*n* = 25) and in the admixed varieties (*n* = 44). Chlorotype names are given according to Arroyo-García et al. [7]

Multiple regression analyses were run to evaluate the effect of this stratification on the nine considered traits (Additional file 6). Moderate and significant (*P* ≤ 0.001) effects were detected for the four berry traits considered, whereas larger effects for bunch length, width and weight were observed, especially for 2013 data, when more than 40 % of phenotypic variance for these bunch traits was explained by the population structure. No significant effect on the number of seeds per berry was observed, whereas the number of berries per bunch was only significantly related in 2011.

Altogether, STRUCTURE results were considered as appropriate and capable to correct for most of spurious associations, so membership coefficients were included in the association tests.

### *VvNAC26* polymorphisms

**Fig. 2:**
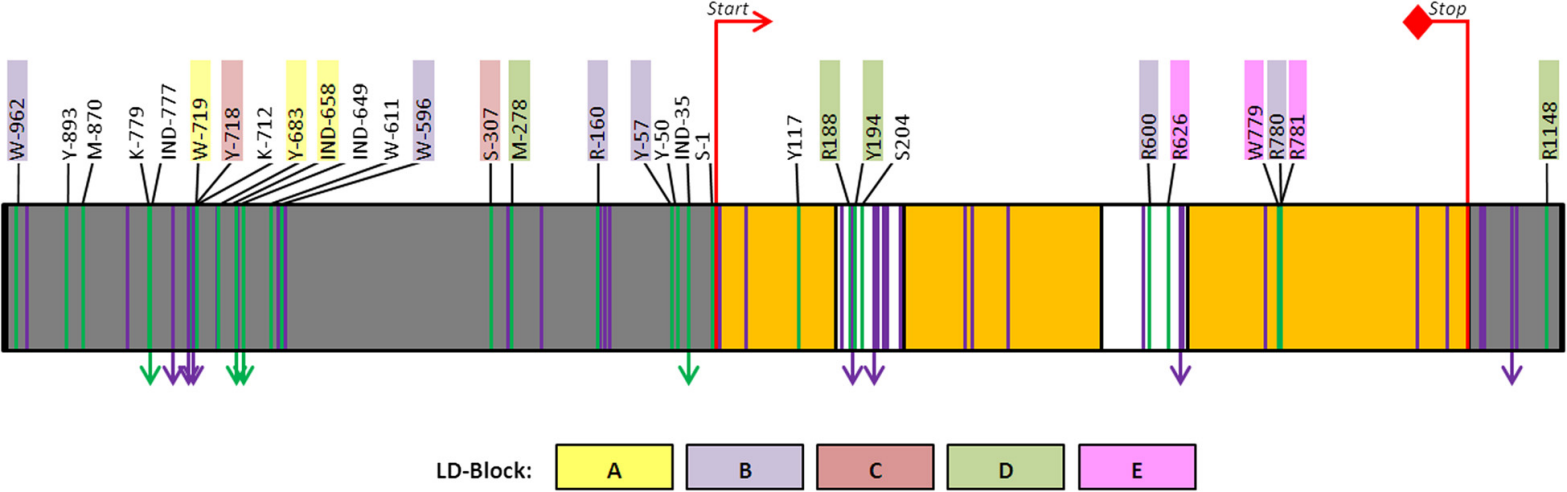
Sequence polymorphisms detected for the *VvNAC26* gene in the 114 grapevine varieties analyzed. SNPs are indicated as vertical lines, whereas INDELs are indicated as vertical arrows. Their color indicates the Minor allele frequency (MAF): violet < 5 %; green >5 %. Only the name of polymorphisms with a MAF > 5 % is specified, for the whole list the reader is referred to the Additional file 7. Red lines indicate ATG-start and STOP codons. Grey boxes indicate promoter and 3’-UTR, whereas orange and white boxes indicate coding regions of exons and introns, respectively. Polymorphisms in the LD-blocks A, B, C, D and E are indicated according to color code

A total of 2184 bp of the *VvNAC26* gene, including 1000 bp of the promoter region, were sequenced in the 114 grapevine varieties. Sequencing and alignment results showed a 100 % coverage (min 20 reads; 93.8 % of sequence over 80 reads; average coverage depth: 117.5 ± 16.7) in all the grapevine varieties. Data can be accessed at NCBI’s Sequence Read Archive (SRA) under the accession code SRP057099. The locus structure annotated for the PN40024 reference genome [60] in the database hosted at CRIBI (12X V1) consisting in three exons (166, 281 and 402 bp), two introns (98 and 106 bp) and a 3’-UTR of 131 bp was identifiable by visual inspection of the aligned reads in the IGV browser and it was further verified by RNAseq analysis (data not shown). Nucleotide sequence analysis enabled the identification of 69 polymorphisms (58 SNPs and 11 INDELs) for the set of varieties considered in this work: 35 polymorphisms were found in the promoter region, 12 in coding regions, 16 in intronic regions, and 6 in the 3’-UTR (Fig. 2 and Additional file 7). Among them, 39 polymorphisms (56.5 %) were represented by a rare allele (minor allele frequency, MAF ≤ 5 %) (Fig. 2 and Additional file 7), most of them exclusively found in the three interspecific hybrids included in our study. As expected, polymorphism density was higher in non-coding regions than in coding regions (in average, one polymorphism every 19.6 nucleotides and every 71.7 nucleotides, respectively). No INDELs were detected in coding regions, being mostly found in the gene promoter. Their length varied considerably, from the IND-35 that involves the insertion/deletion of 11 nucleotides to events involving a unique nucleotide (IND-745, IND-717, IND-658, IND-649, IND643 and IND1100). Among the 58 detected SNPs, 3 were found in the first exon, 3 in the second exon, and 6 in the coding portion of the third exon. Four of them caused non-synonymous changes in the corresponding amino acid [S405 (Ala/Pro), R761 (Asp/Gly), W779 (Gln/Leu), and R781 (Val/Met)]. According to SNAP and PROVEAN results, none of them would generate a non-neutral effect on the function of the protein (Additional file 7).

LD analysis revealed the presence of five blocks of polymorphisms in high level of LD (*r*^2^ ≥ 0.8, *P* ≤ 0.001): LD-block A (comprising three SNPs: W-719, Y-683 and IND-658), LD-block B (six SNPs: W-962, W-596, R-160, Y-57, R600 and R780), LD-block C (two SNPs: Y-718 and S-307), LD-block D (four SNPs: M-278, R188, Y194 and R1148), and LD-block E (three SNPs: R626, W779 and R781) (Fig. 2 and Additional file 8).

### *VvNAC26* haplotypes

On the basis of the 69 polymorphisms detected (Additional file 7), the PLEM algorithm [71] implemented in PHASE inferred 26 different haplotypes, including 9 unique haplotypes (present in 1 variety, frequency 0.4 %) (Table 3). None of the algorithms used in the RDP4 software indicated any evidence of recombination in the 26 haplotypes. Only four haplotypes (H3, H17, H19 and H20) showed a frequency ≥5 %, accounting for 72.8 % of the haplotypes in the grapevine varieties analyzed. H3 was exclusively found in varieties of the *k3* genetic group or in admixed varieties; H17 was found in the three groups, with a major presence in *k1* and *k3*; H19 was found only in *k1* and *k2*; and H20 was found in varieties assigned to any of the genetic groups (Table 3). Only four different haplotypes were found in the 10 varieties attributed to the *k2* group (H8, H17, H19 and H20) (Table 3), with four table grape varieties (Italia, Cardinal, Paraiso and Afus Ali) being homozygous for the haplotype H20 (Additional file 1).

**Table 3:** *VvNAC26* haplotypes (H1-H26)

H	Haplotype	Global population	k1	k2	k3
H1	TTTCAT010AC1GCT1TT1TGTTGACAAAAACC1CACCTG0CAG0CCTCAGGAAG0TAAGGCGGTG1TG	1 (0.4 %)	-	-	-
H2	TTTCAT110AC1GCT1TT1TGTTGACAAAAACC1CACCTG0CAG0CCTCAGGAAG0TAAGGCGGTG1TG	5 (2.2 %)	-	-	4 (8.0 %)
H3	TTTCAT110AC1GCT1TT1TGTTGAAAAAAACC1CACCTA1TAG0CCTCAGGAAA1TATGACGGTG0TA	13 (5.7 %)	-	-	8 (16.0 %)
H4	TTTAGT110TT1GCC0TT1TGTTCACAAGGACC1CACCTG1CAG0CTTCAGGAAG1TAAGGCGGTG0TG	8 (3.5 %)	1 (1.4 %)	-	3 (6.0 %)
H5	TTTAAT010AC1GCT1TT1TGTTGACAAAAACC1CACCTG1CAC0GCTCAGGGAG1TAAGGCGGTG0TG	1 (0.4 %)	-	-	1 (2.0 %)
H6	TTTAAG010TT1GCC0TT1AGTTCACAAAAACC1CACCTG1CAG0CCGTAGGAAA1CATGACGGTG0TG	3 (1.3 %)	3 (4.3 %)	-	-
H7	TTTAAG010AC1GCT1TT1TGTTGACAAAAACC1CACCTG1CAC0CCTCAGGAAA1TATGACGGTG0TG	2 (0.9 %)	-	-	2 (4.0 %)
H8	TTTAAG010AC1GCT1TT1TGTTGACAAAAACC1CACCTG1CAC0GCTCAGGGAG1TAAGGCGGTG0TG	4 (1.8 %)	-	1 (5.0 %)	2 (4.0 %)
H9	TTCAAG010TT1GCC0TT1AGTTCACAAAAACC1CACCTG1CAC0CCTCAGGAAA1TATGACGGTG0TG	10 (4.4 %)	4 (5.7 %)	-	1 (2.0 %)
H10	TTCAAG010TT1GCC0TT1AGTTCACAAAAACC1GACCTG1CAC0CCTCAGGAAA1TATGACGGTG0TG	1 (0.4 %)	-	-	-
H11	TTCAAG010TT1GCC0TT1AGTTCACAAAAATC1CACCTG1CAC0CCTCAGGAAA1TATGACGGTG0TG	1 (0.4 %)	-	-	-
H12	TTCAAG010AT1GCT1TT1TGTTCACAAAAACC1CACCTG1CAC0CCTCAGGAAA1TAAGGCGGTG0TG	1 (0.4 %)	-	-	-
H13	TTCAAG010AC1GCT1TT0TGTTGAAAAAAACC1CACCTG1TAG0GCTCAGCGAG1TAAGGCGGTG0TG	2 (0.9 %)	-	-	2 (4.0 %)
H14	TTCAAG011AT1GCC1TT0TCTACACAAAAACT1CGTCTG1CCG0GCTCAGGAAA1TAAGATGACG0TG	1 (0.4 %)	-	-	-
H15	TTCAAG000AC1GCT1TA1TGTTCACAAAAACC1CACCTG1CAC0CCTCAGGAAA1TAAGGCAGTG0CG	3 (1.3 %)	1 (1.4 %)	-	-
H16	TCCAAT010AT0GTT1AT1TCTTCGCCAAAGCT1GACCTG1CAC0CCTCTCGAAA1TATGACGGTA0TG	2 (0.9 %)	-	-	1 (2.0 %)
H17	ATCAAT010AT1GCT1TT1TGATCACAGAAATT1GACCTG1CAC0CCTCAGGAGG1TAAAGCGGTG0TG	86 (37.7 %)	31 (44.3 %)	4 (20.0 %)	16 (32.0 %)
H18	ATCAAT010AT1GCT1TT1TGATCACAGAAATT1GACTTG1CAC0CCTCAGGAGG1TAAAGCGGTG0TG	1 (0.4 %)	1 (1.4 %)	-	-
H19	ATCAAT010AT1GCT1TT1TGATCACAGAAATT0GACTTG1CAC0CCTCAGGAGG1TAAAGCGGTG0TG	14 (6.1 %)	6 (8.6 %)	1 (5.0 %)	-
H20	ATCAAT010AT1GCT1TT0TGATCACAGAAATT1GACTTG1CAC0CCTCAGGAGG1TAAAGCGGTG0TG	53 (23.2 %)	19 (27.1 %)	14 (70.0 %)	7 (14.0 %)
H21	ATCAAT010AT1GCT0TT0TGATCACAGAAATT1GACTTG1CAC0CCTCAGGAGG1TAAAGCGGTG0TG	2 (0.9 %)	-	-	-
H22	ATCAAT010AT1TCT1TT1TGATCACAGAAATT1GACCTG1CAC0CCTCAGGAGG1TAAAGCGGTG0TG	4 (1.8 %)	1 (1.4 %)	-	1 (2.0 %)
H23	ATCAAT010AT1TCT1TT1TGATCACAGAAATT1GACCTG1CAC0CCTCAGGAGG1TGAAGCGGTG0TG	1 (0.4 %)	-	-	1 (2.0 %)
H24	ATCAAT010AT1TCT1TT1TGATCACAGAAATT1GACCCG1CAC0CCTCAGGAGG1TAAAGCGGTG0TG	6 (2.6 %)	3 (4.3 %)	-	-
H25	ATCAAT010AT1TCT1TT1TGATCACAGAAATT1GACCCG1CAC1CCTCAGGAGG1TAAAGCGGTG0TG	2 (0.9 %)	-	-	-
H26	ATCAAT110AT1GCT1TT1TGATCACAGAAATT1GACCTG1CAC0CCTCAGGAGG1TAAAGCGGTG0TG	1 (0.4 %)	-	-	1 (2.0 %)

Their absolute (*n*) and relative (%) frequencies are given for the global population (*n* = 114) and the genetic groups established by STRUCTURE [*k1* (*n* = 35), *k2* (*n* = 10), and *k3* (*n* = 25)]. INDELs are coded as 1/0 for insertion/deletion events, respectively

The diversity parameters and neutrality tests calculated for the *VvNAC26* gene sequence in the whole set of varieties and in the three genetic groups are shown in Additional file 9. Nucleotide diversity (π) and Watterson’s estimate (θ) released values of 0.00657 and 0.00825 (respectively) for the 26 haplotypes found in the whole collection. Group *k2* obtained lower values of diversity than *k1* and *k3*, probably due to the lower number of haplotypes (4) and polymorphic sites (17) found in this group. Tajima’s *D* and Fu and Li’s D* tests were not significant in either the global collection or the three genetic groups (Additional file 9).

**Fig. 3:**
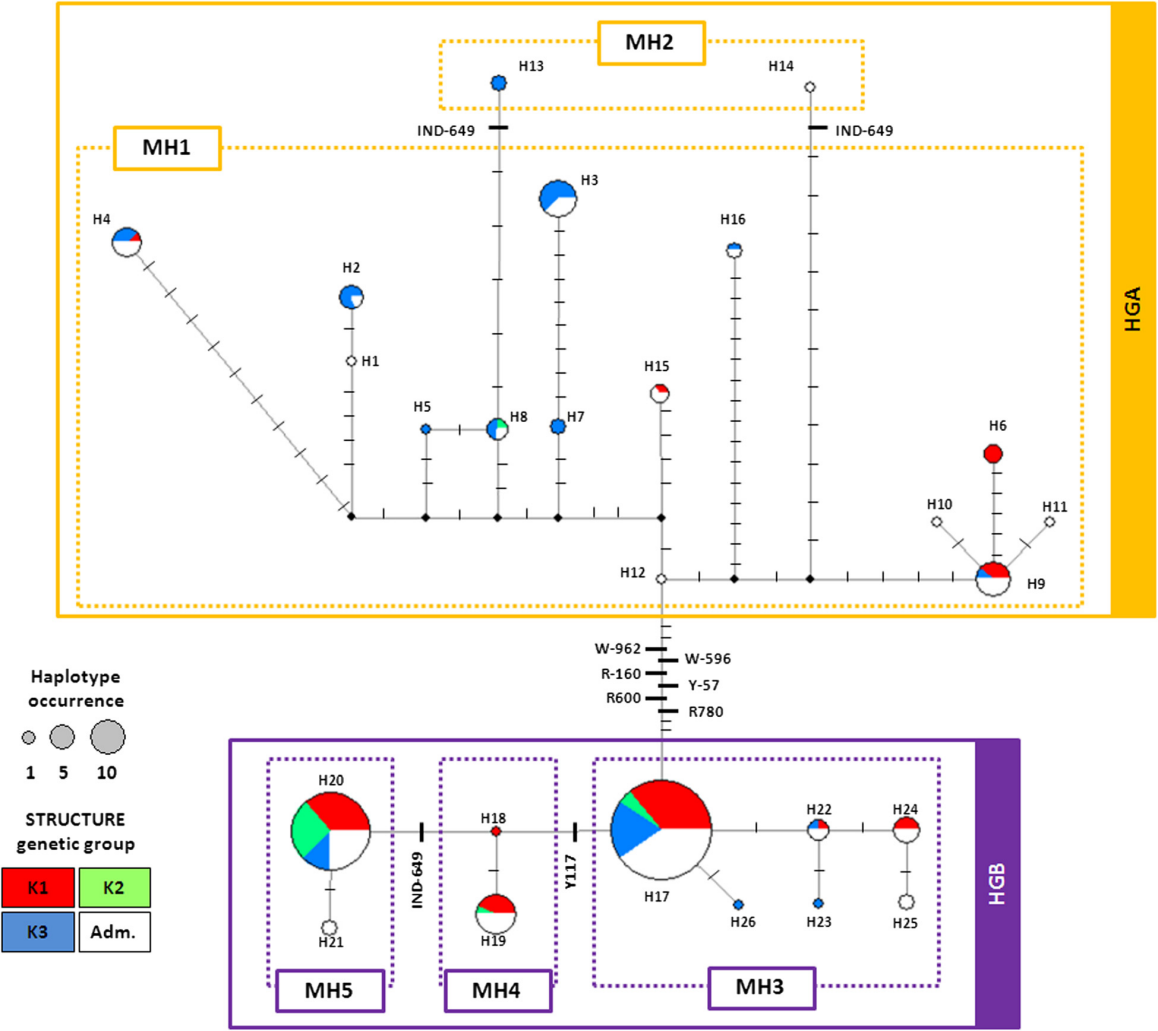
Median-joining phylogenetic network constructed for the 26 *VvNAC26* haplotypes detected (H1 – H26). Each haplotype is represented by a circle, which size (see code) is proportional to its frequency in the set of varieties analyzed. Their inner color/s indicate the proportion of varieties assigned to each of the genetic groups detected by STRUCTURE (see color code, Adm.: admixed). Lines connecting haplotypes represent phylogenetic branches, and small transversal lines represent mutational steps (only those polymorphisms significantly associated with berry and/or bunch traits appear named, according to Table 4). Black dots represent missing intermediate haplotypes. HGA and HGB indicate the two different haplogroups detected (see Additional file 10). MH1, MH2, MH3, MH4 and MH5 indicate the different minihapolotypes inferred on the basis of polymorphisms Y117, W-962 and IND-694 (see Table 5)

The hierarchical clustering of *VvNAC26* haplotypes based on Ward’s method revealed the presence of two groups of haplotypes (or haplogroups, HG): HGA, comprising 16 haplotypes (accounts for 25.4 % of the haplotype abundance in the set of varieties considered) and HGB, with the remaining 10 haplotypes (Additional file 10A). Accordingly, haplotype network discriminated these two haplogroups (Fig. 3), which differed in ten SNPs (W-962, K-779, W-592, R-160, Y-57, Y-50, S-1, R600, R626 and R780), mostly of the LD-block B (Additional file 8). The other detected LD-blocks are in minor branches of the network (data not shown), so they are not further discussed. Considering the distribution of the haplotypes in the three genetic groups, haplogroup HGA includes haplotypes mainly present in wine varieties of groups *k1* and *k3*; only one variety assigned to the *k2* genetic group (Barbera Nera, an Italian wine variety) was found to have a HGA haplotype (H8) (Additional file 1). The haplogroup HGA contains one of the most abundant haplotypes -H3- exclusively found in varieties assigned to *k3* (Fig. 3 and Table 3). Haplotypes in HGB were well distributed within the varieties assigned to the three genetic groups *k1* (35.9 %), *k2* (11.2 %) and *k3* (15.3 %). This haplogroup contained the other three most abundant haplotypes found in the set of varieties analyzed (H17, H19 and H20, Fig. 3). As mentioned above, H20 was commonly found in the grapevine varieties assigned to the group *k2* (Fig. 3).

### Association tests

We found eight polymorphisms significantly associated with different berry and bunch traits with a *P*-value below the established threshold of 6.53E^-3^. One of them still showed statistical significance when considering the more stringent threshold (3. 27E^-4^) (Table 4).

**Table 4:** *VvNAC26* polymorphisms showing significant associations with berry and bunch traits

Polymorphism	LD-Block	Trait	2011		2012		2013	
			*P*-value	R^2 ^(%)	*P*-value	R^2^(%)	*P*-value	R^2^ (%)
W-962	B	Berry length	6.40E^-3^*	8,44	2.16E^-3^*	9,38	5.74E^-4^*	12,28
		Berry volume	2.78E^-2^	6,03	3.79E^-3^*	8,66	2.15E^-3^*	8,95
		Berry weight	2.43E^-2^	6,21	5.55E^-3^*	8,10	2.41E^-3^*	8,74
		Berry width	2.31E^-2^	6,40	3.65E^-3^*	8,79	3.89E^-3^*	8,69
IND-649	-	Berry length	2.74E^-2^	5,91	2.20E^-3^*	9,35	1.20E^-2^	7,04
		Berry volume	2.97E^-2^	5,91	2.03E^-3^*	9,69	1.09E^-2^	6,46
		Berry weight	2.79E^-2^	5,98	2.06E^-3^*	9,74	1.04E^-2^	6,52
		Berry width	2.08E^-2^	6,59	6.42E^-4^*	11,73	2.36E^-2^	5,97
		Bunch weight	1.11E^-1^	3,23	4.44E^-2^	4,71	4.55E^-3^*	6,95
W-596	B	Berry length	6.40E^-3^*	8,44	2.16E^-3^*	9,38	5.74E^-4^*	12,28
		Berry volume	2.78E^-2^	6,03	3.79E^-3^*	8,66	2.15E^-3^*	8,95
		Berry weight	2.43E^-2^	6,21	5.55E^-3^*	8,10	2.41E^-3^*	8,74
		Berry width	2.31E^-2^	6,40	3.65E^-3^*	8,79	3.89E^-3^*	8,69
R-160	B	Berry length	6.40E^-3^*	8,44	2.16E^-3^*	9,38	5.74E^-4^*	12,28
		Berry volume	2.78E^-2^	6,03	3.79E^-3^*	8,66	2.15E^-3^*	8,95
		Berry weight	2.43E^-2^	6,21	5.55E^-3^*	8,10	2.41E^-3^*	8,74
		Berry width	2.31E^-2^	6,40	3.65E^-3^*	8,79	3.89E^-3^*	8,69
Y-57	B	Berry length	6.40E^-3^*	8,44	2.16E^-3^*	9,38	5.74E^-4^*	12,28
		Berry volume	2.78E^-2^	6,03	3.79E^-3^*	8,66	2.15E^-3^*	8,95
		Berry weight	2.43E^-2^	6,21	5.55E^-3^*	8,10	2.41E^-3^*	8,74
		Berry width	2.31E^-2^	6,40	3.65E^-3^*	8,79	3.89E^-3^*	8,69
Y117	-	Berry length	2.95E^-4^**	14,04	7.43E^-5^**	15,05	7.41E^-4^*	11,83
		Berry volume	1.26E^-4^**	16,03	1.27E^-5^**	18,59	1.33E^-3^*	9,70
		Berry weight	1.18E^-4^**	16,03	2.50E^-5^**	17,48	1.28E^-3^*	9,73
		Berry width	6.20E^-5^**	17,57	2.58E^-6^**	21,75	7.32E^-4^*	11,51
		Bunch length	3.94E^-3^*	8,55	9.68E^-3^	6,90	9.73E^-3^	6,01
		Bunch weight	3.71E^-4^*	12,39	7.20E^-3^	7,61	7.54E^-4^*	9,46
R600	B	Berry length	6.40E^-3^*	8,44	2.16E^-3^*	9,38	5.74E^-4^*	12,28
		Berry volume	2.78E^-2^	6,03	3.79E^-3^*	8,66	2.15E^-3^*	8,95
		Berry weight	2.43E^-2^	6,21	5.55E^-3^*	8,10	2.41E^-3^*	8,74
		Berry width	2.31E^-2^	6,40	3.65E^-3^*	8,79	3.89E^-3^*	8,69
R780	B	Berry length	6.40E^-3^*	8,44	2.16E^-3^*	9,38	5.74E^-4^*	12,28
		Berry volume	2.78E^-2^	6,03	3.79E^-3^*	8,66	2.15E^-3^*	8,95
		Berry weight	2.43E^-2^	6,21	5.55E^-3^*	8,10	2.41E^-3^*	8,74
		Berry width	2.31E^-2^	6,40	3.65E^-3^*	8,79	3.89E^-3^*	8,69

*P*-values of associations and variance explained by the marker (R^2^) are indicated for the MLM models obtained for 2011, 2012 and 2013

*P-value ≤ 6.53E^-3^; **P-value ≤ 3.26E^-4^

Six SNPs located in the LD-block B (W-962, W-596, R-160, Y-57, R600 and R780) showed a significant association with berry length, volume, weight and volume, explaining up to 12.28 % of berry length variation in 2013 (Table 4). As stated before, the LD-block B was located in the phylogenetic branch differentiating HGA and HGB (Fig. 3).

Y117 - a synonymous SNP located in the first exon of *VvNAC26* (Fig. 2 and Additional file 7) - showed to be significantly associated with berry width, length, weight and volume, as well as with bunch length and weight (*P* ≤ 6.53E^-3^). *P*-values obtained for associations with berry length, volume weight and width in 2011 and 2012 were significant even when considering the more stringent threshold (3. 27E^-4^). The strongest association found was between Y117 and berry width in 2012 (*P* = 2.58E^-6^), and the marker explained up to 21.7 % of trait variance (Table 4). In the phylogenetic network, this SNP was found in the haplogroup HGB, in the branch separating H17 from H18 (Fig. 3).

Indel IND-649, located in the promoter region, was also significantly associated with berry length, volume, weight and width in 2012 and bunch weight in 2013 (*P* ≤ 6.53E^-3^) (Table 4). IND-649 was found in different positions in the network constructed for the 26 *VvNAC26* haplotypes (Fig. 3). Specifically, it was found in the phylogenetic branch separating H20 from H18 in haplogroup HGB, as well as in the HGA haplogroup, in the branches separating H13 from H8 and H14 from H12. As stated above, IND-649 involves the insertion/deletion of a unique nucleotide, and it was found to be located in a poly-T region, so the variation in this position leads to a (T)_9_ or (T)_10_ genotype. Alleles found in H13, H14, H20 and H21 are identical in size for this locus [(T)_9_] but in the network they do not derive from a common ancestor, which may reflect size homoplasy in this site.

As commented above, the automatic prediction carried out by means of SnpEff [65] revealed that SNP Y117 does not affect the primary structure of the protein (Additional file 7), and the mRNA structure analyses using two independent tools [68, 69] predict that Y117 does not induce any structural change in its secondary structure (Additional file 11). Based on the SnpEff [65] and PlantCARE [70] results, only one SNP (W-962) of the LD-block B would be located in a regulatory region (a CAAT-box). Similar *in silico* analysis revealed that IND-649 is located in a TATA-box, suggesting the possible regulatory effect of both polymorphisms in *VvNAC26* expression.

### Associated polymorphisms define minihaplotypes associated with berry size

Single-marker associations and LD suggest that W-962 (representing the associated LD-block B), IND-649 and Y117 contribute particularly to the relationship found between *VvNAC26* and berry traits, as well as to the phylogenetic clustering of the inferred haplotypes. In fact, the hierarchical clustering of the 26 haplotypes using only these three polymorphic sites is similar to that obtained when using the 69 polymorphisms, denoting their relevance in the clustering (Additional file 10A and B). To evaluate their joint effect on berry size, we used W-962, IND-649 and Y117 to infer a reduced set of polymorphism combinations (minihaplotypes, MH) for a haplotype-based association analysis, which has been suggested as a more powerful approach since it considers the underlying LD between different polymorphic sites [71, 95, 96]. Out of the eight possible theoretical combinations, we found five different minihaplotypes in the set of varieties analyzed (Table 5). They have variable frequencies in our set of grapevine varieties, with values ranging from 1.3 % (MH2) to 43.9 % (MH3), and they are unevenly distributed in the three genetic groups established by STRUCTURE: MH3 was the most abundant in the group *k1* (50 %), MH5 in *k2* (70 %) and MH1 (44 %) and MH3 (38 %) in *k3* (Table 5). Minihaplotypes MH1 and MH2 were found in the haplogroup HGA, whereas MH3, MH4 and MH5 were found in HGB (Fig. 3). Thus, minihaplotypes were used for another association analysis, excluding MH2 due to its low frequency. They were also significantly associated with berry dimensions in 2011, 2013 and 2013 (Table 6). The percentage of variance of the different traits explained by the minihaplotypes is higher than those explained by any of the individual polymorphisms (Table 4), suggesting an additive effect of these three markers in the phenotype of the berry.

**Table 5:** *VvNAC26* minihaplotypes (MH) constructed from the combination of three polymorphisms (W-962, IND-649 and Y117)

	W-962	IND-649	Y117	Global population	k1	k2	k3
MH1	T	ins	C	55 (24.1 %)	9 (12.9 %)	1 (5.0 %)	22 (44.0 %)
MH2	T	del	C	3 (1.3 %)	-	-	2 (4.0 %)
MH3	A	ins	C	100 (43.9 %)	35 (50.0 %)	4 (20.0 %)	19 (38.0 %)
MH4	A	ins	T	15 (6.6 %)	7 (10.0 %)	1 (5.0 %)	-
MH5	A	del	T	55 (24.1 %)	19 (27.1 %)	14 (70.0 %)	7 (14.0 %)

Their absolute (*n*) and relative (%) frequencies are shown for the global population and the three genetic groups established by STRUCTURE [*k1* (*n* = 35), *k2* (*n* = 10), and *k3* (*n* = 25)]

**Table 6:** *VvNAC26* minihaplotype-based association results

Trait	2011		2012		2013	
	*P*-value	R^2^(%)	*P*-value	R^2^(%)	*P*-value	R^2^(%)
Berry length	2.34E^-3^*	20,0	1.95E^-3^*	20,2	1.49E^-3^*	22,2
Berry volume	2.42E^-3^*	20,9	1.25E^-3^*	21,7	5.85E^-3^	17,4
Berry weight	2.55E^-3^*	20,6	1.76E^-3^*	21,0	5.38E^-3^*	17,5
Berry width	2.12E^-3^*	21,1	1.87E^-3^*	26,7	6.20E^-3^	18,0

*P*-values and explained variance of the marker (R^2^) for the MLM models obtained between the berry traits included in this work and the minihaplotypes defined by the combination of three *VvNAC26* polymorphisms (W-962, IND-649 and Y117)

*P-value ≤ 5.55E^-3^ (Bonferroni-corrected threshold for multiple comparisons for α = 0.05)

### Phenotypic values related to associated markers and minihaplotypes

**Fig. 4:**
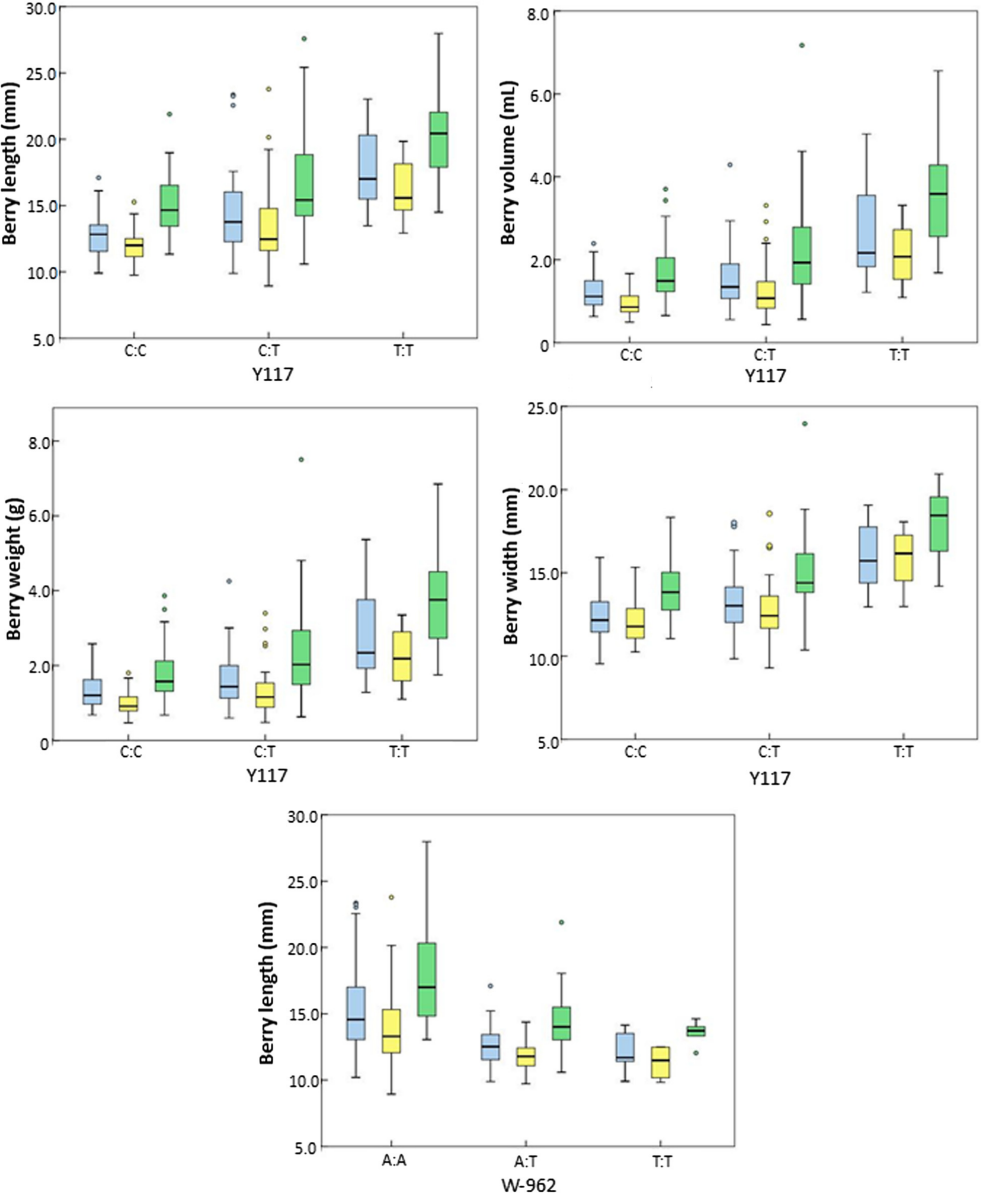
Berry phenotypes for the different Y117 and W-962 genotypes. Bow-plots are only shown for those marker/traits associations recursively found in 2011 (blue), 2012 (yellow) and 2013 (green) (see Table 4). Outliers are indicated as circles

As seen before, Y117 showed to be associated with the size of the berry (Table 4). The minor allele of this polymorphism (T) was highly frequent in the grapevine collection used (30.7 %) (Additional file 7). Homozygous T:T varieties tend to produce larger berries than the heterozygous C:T and the homozygous C:C genotypes, which have similar berry dimensions in average (Fig. 4). In the same way, Y117 was associated with bunch weight and length, with the grapevine varieties containing two T alleles more prone to produce heavier and longer bunches than the other genotypes (Fig. 4). Similarly, homozygous individuals for the A allele at the SNP W-962 (selected for representing the LD-block B) tend to have bigger berries than those at heterozygous or homozygous states for the minor allele T, which showed a similar phenotype (Fig. 4). This minor allele was highly present in the grapevine collection (25.4 %). Finally, the deletion event at IND-649 (present in 25.4 % of the set of varieties) was associated with larger berries and heavier bunches (data not shown).

**Fig. 5:**
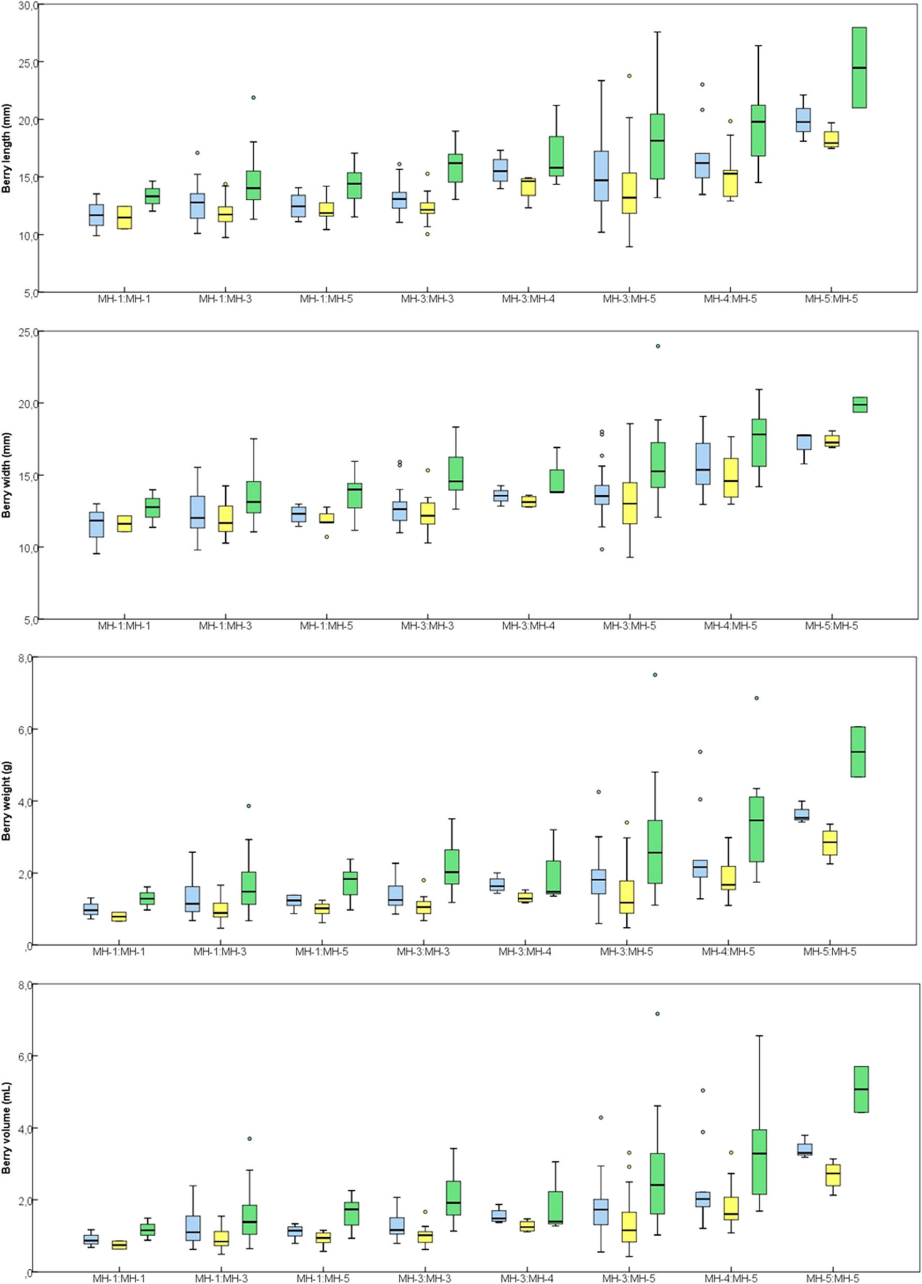
Berry phenotype (length, width, volume and weight) for the different minihaplotype (MH) pairs detected. Minihaplotypes were inferred on the basis of three selected polymorphisms (Y117, W-962 and IND-694). Box-plots are shown for 2011 (blue), 2012 (yellow) and 2013 (green). Outliers are indicated as circles

Phenotypic effects were also observed when considering the minihaplotypes built through the combination of these three polymorphic sites. Accordingly, varieties carrying in homozygosis the T allele at Y117, the A allele at W-962 and the deletion [(T)_9_] at IND-649 (so MH5:MH5 varieties) showed the largest berries within the set of varieties evaluated (Fig. 5). As mentioned above, this minihaplotype was the most common one in the group *k2* (Table 5), characterized for including most of the *orientalis* table grape varieties considered in this work (Additional file 1). By contrast, homozygous individuals for the minihaplotype MH1, that combines the C allele at Y117, the T allele at W-962 and the allele with the insertion [(T)_10_] at IND-649 (Table 5), showed the smallest berries (Fig. 5). This minihaplotype was commonly found in *k3* (Table 5), a group mostly composed by *occidentalis* European wine varieties of small-sized berries (Additional file 1). Heterozygous individuals carrying both minihaplotypes (MH1:MH5) showed a similar phenotype than the homozygous individuals for the MH1 minihaplotype (MH1:MH1) (Fig. 5).

## Discussion

Berry size depends on many genetic, developmental and environmental factors, including specific pre-anthesis flower features and multiple post-pollination events [11, 97]. In Arabidopsis, the NAC domain containing protein NAP gene has been reported to be involved in multiple developmental processes, from the establishment of flower meristem identity and flower organ formation to fruit ripening and senescence [38, 51, 98]. A role in flower and berry development has been suggested for *VvNAC26* [52], the grapevine NAP homolog [50], on the basis of its gene expression profile. As stated before, several QTL for berry size have been reported [17–22], but none of them in the region where *VvNAC26* is located. This could be due to the fact that the progenies studied arise from crosses involving only wine or only table cultivars. *VvNAC26* was chosen as a candidate gene that has been sequenced in a set of varieties to determine the existing nucleotide variation, and to identify its possible contribution to the natural variation observed for several reproductive traits in grapevine.

A relatively high rate of nucleotide variation was found for *VvNAC26* in the grapevine varieties considered, with an average of one polymorphic site every 31 nucleotides. This variation is higher than the reported in other studies that included non-*vinifera* individuals for the analysis of the nucleotide variation of different grapevine genes [99, 100]. Nonetheless, these works do not include the analysis of the promoter region, where we found a high number of polymorphic sites. The analysis of these regulating regions is paramount in association genetics surveys, since different variants in the gene promoter may correlate with different expression level and, ultimately, phenotypic diversity [101]. On the other hand, some of the rare polymorphisms detected in the *VvNAC26* sequence were only found in the three interspecific hybrids included in this study, and they are likely attributable to their non-*vinifera* genetic background. As expected, we found a higher mutation rate in non-coding regions than in coding regions [102], and only twelve polymorphisms were detected in exonic regions. Four of them generated amino acid substitutions, although they are predicted to be neutral in the protein. As a result there is a high degree of conservation of the VvNAC26 protein in the cultivated grapevine. A high level of conservation was also reported for another grapevine NAC protein (VvNAC4), with only one non-synonymous SNP detected in the gene sequence of 50 wild accessions and 73 cultivars [100]. Average intragenic LD calculated for all pairs of polymorphic sites with frequency over 5 %, was 0.25, similar to the average LD value reported for the *VvMybA1* gene [27]. Six blocks of polymorphisms in high LD were identified in the *VvNAC26* sequence and, as for other grapevine genes [28, 32], some of those polymorphisms were found in high LD despite being largely separated in the nucleotide sequence.

The LD-block B separates the two main haplogroups (HGA and HGB) detected in the sequenced samples, and thus these polymorphisms could be related to ancestral alleles. Considering our set of grapevine varieties and according to the phylogenetic network and the hierarchical clustering of the *VvNAC26* haplotypes, HGA and HGB show important differences. HGA includes 16 haplotypes found in low frequency in the global population studied, which are very divergent regarding the high number of polymorphisms found in this group, but very uniform in terms of their use and berry size (wine varieties/small berries). On the other hand, HGB includes 10 haplotypes, genetically closer (less polymorphisms), and that are found indistinctly in wine and table varieties with diverse berry size.

A positive relationship between haplotype frequency and antiquity has been proposed [99]. Considering that haplotype H17 (in HGB) presents the highest frequency in our sample, it could be suggested as the most ancestral one within the haplotypes detected, which is supported by the fact that the oldest known varieties, such as Pinot Noir, or Traminer, bear an H17 haplotype. H17 is a good candidate to have been the target of mutation/selection events during early domestication and selection processes. The varieties with this haplotype are currently used either for wine or for both wine and table, and have a low-medium berry size, so they are of the wine (*occidentalis*) or intermediate (*pontica*) morphotypes. But, at the same time, this haplotype H17 is only two mutations far from H20, characteristic of table grapes with large berries (*orientalis* morphotype). Thus, it can be hypothesized that, starting from H17, the selection of genotypes carrying mutations for SNP Y117 (recurrently associated with berry length, width, volume and weight in 2011, 2012 and 2013) and INDEL IND-649 (associated with berry dimensions in 2012) generated a largest berry size and were thus favored in table grape cultivars. On the contrary, genotypes mutated for the LD-block B polymorphisms (associated with berry length in 2011, 2012 and 2013 and berry volume, weight and width in 2012 and 2013, and discriminating HGA and HGB groups) generated the smallest berries, being likely preferred for the development of wine grape cultivars.

Individual polymorphisms may cause relevant changes in gene expression or in protein function, which may ultimately cause alterations in a certain phenotype. However, polymorphisms are not inherited individually, but in LD with other genetic variants, in which certain alleles of close polymorphisms are found together. Consequently, the combination of some polymorphisms in minihaplotypes may have an stronger biological effect that single markers [95]. Consistent with the association results for the individual markers, the minihaplotype-based association analyses also released significant associations with berry traits. Homozygous individuals for the minihaplotype MH5 showed the biggest berries within the set of analyzed varieties, and all of them are mostly grown for the production of table grapes. Very interestingly, they present different chloroplast haplotypes (Afus Ali: A; Cardinal: B; Italia: C; Paraíso: D), indicating that they have different genetic origins (at least for the maternal lineage), and that this minihaplotype has been selected for table grape production in different genetic backgrounds. In this light, we analyzed the *VvNAC26* sequence of cv. Red Globe, a highly appreciated table grape variety characterized by its very big berry size. It has no close relationship with the large-berried varieties studied here, and it is also homozygous for the MH5 genotype (data not shown), supporting the role of this minihaplotype in the berry size, independently of its genetic origin.

Putative functional effects of the three polymorphisms associated with berry size (W-962, IND-649 and Y117) are likely not related to the activity of the encoded protein. SNP W-962 (in LD-block B) and IND-649 are not located in the coding region, but in two common *cis*-regulatory elements. On the other hand, Y117 is a synonymous mutation, and *in silico* predictions showed no structural differences in the *VvNAC26* mRNAs encoded by both variants in Y117. So, no effect in the stability and conformation of the transcribed *VvNAC26* mRNA is expected, which might have affected critical post-transcriptional processes [103]. Considering the long intragenic LD observed for several polymorphic sites within *VvNAC26*, Y117 could be in LD with an undetected polymorphism responsible for trait variation [104], regulating gene expression and located outside the sequenced region. This situation has been previously suggested to explain the effect of a silent polymorphism of *VvGAI1* associated with berry texture [30]. In fact, Clark et al. [105] confirmed the role of a *cis*-acting enhancer located between 41 and 69 kb upstream from the maize *teosinte branched1* (*tb1*) gene starting site as the main causative factor controlling *tb1* expression and *tb1*-related phenotypes. According to our results, it seems likely a functional effect of the *VvNAC26* polymorphisms associated to berry size related to the regulation of gene transcription. Further analyses aimed at evaluating *VvNAC26* expression levels in key stages of pistil and berry development in the extreme genotypes found (e.g.: MH1:MH1, MH1:MH5 and MH5:MH5) may yield additional information on the role of this gene and the associated polymorphisms in the final berry size. Consistently with the likely regulatory role of the associated polymorphisms, differential expression of *VvNAC26* (=*VvNAP*) correlated with differential berry development and growth in the grapevine *flb* somatic variant (bearing fleshless berries), compared to the wild type Fernandez et al. [52]. In this somatic variant, high expression of *VvNAP* correlated with reduced berry growth. Indeed, Arabidopsis mutants over-expressing *NAP* showed a reduced size of several floral organs [38]. Altogether, these results suggest that the larger berry size observed for certain *VvNAC26* variants might be a consequence of a reduced gene expression.

Analysis of *VvNAC26* in the expression atlas developed for cv. Corvina [106] shows that, as seen for Arabidopsis *NAP* [38], *VvNAC26* expression is not only related to *VvPI* expression (Additional file 12). In this line, a high expression of *VvNAC26* is also appreciated in many other tissues, including senescing and mature tissues (Additional file 12) [106], in agreement with the promotion of senescence that have been proposed for *NAP*-like genes in Arabidopsis and other species [107–109]. Recent reports indicate that NAP could function via positive regulation of abscisic acid (ABA) biosynthesis [110–112], suggesting that *VvNAC26* could mediate its responses by regulating the expression of ABA-related genes. High levels of ABA have been shown to inhibit cell growth in unpollinated tomato (*Solanum lycopersicum* L.) ovaries, keeping them in a dormant state until pollination [113]. In grapevine, a high level of ABA in flowers at full bloom (coincident with peaks of *VvNAC26* expression, Additional file 12) and high levels of its degradation products after pollination have been reported [114, 115]. Moreover, expression data reported for cv. Moscatel Rosada shows a high down-expression of *VvNECD1* (involved in ABA biosynthesis) in very early pollinated ovaries when compared to the unpollinated ones [116]. These evidences suggest that polymorphisms reducing *VvNAC26* expression might result in lower ABA levels, allowing a greater cell growth rate in ovaries and/or berries which ultimately would give place to larger berries. This hypothesis could be confirmed through analyses aimed at determining ABA levels in flowers and berries at several stages of development in different varieties bearing in homozygous state the extreme *VvNAC26* minihaplotypes identified.

Association results presented here may have a potential limitation given the number of markers used for structure estimation. Thus, further studies aimed to verify these results are needed, using a different set of varieties. Replication of the genetic association study in additional independent samples is the better approach for verifying (or rejecting) associations [117, 118]. Anyway, and considering the suggested role of *VvNAC26* in the early development of grapevine flowers and berries [52], *VvNAC26* and the polymorphisms and minihaplotypes detected in this work (whether causative or a result of allele selection during domestication and selection processes) are good candidates for their further validation prior their use in marker-assisted selection programs aimed to improve fruit size in grapevine breeding programs.

## Conclusions

The analysis of the nucleotide sequence variation at the grapevine *VvNAC26* gene and its association with grapevine reproductive traits has allowed the detection of polymorphisms recurrently associated with berry size. The phylogenetic analysis of the observed *VvNAC26* haplotypes suggests that some of these polymorphisms could have been selected during the development of table grape varieties, given the key importance of the berry size in their use for fresh consumption. The sequence position and predicted functional effects of two associated polymorphisms suggest that they could affect the expression level of *VvNAC26*, what could have an effect on cell growth and berry size. Further analyses evaluating the associated *VvNAC26* polymorphisms*/*haplotypes identified in this work are required to confirm this possibility, and also for using the associated polymorphisms for marker-assisted selection to improve fruit size in grapevine breeding programs.

## Additional files

Additional file 1:
**List of the 114 grapevine varieties evaluated in this study.** (XLSX 24 kb) Additional file 2:
**Phenotypic distribution of the nine traits analyzed in this study for 2011 (skyblue), 2012 (yellow) and 2013 (green).** (PDF 196 kb) Additional file 3:
**Cumulative distribution of the** ***P*** **-values obtained for the trait-marker associations considering a naïve model (blue line) and three models controlling for different type of relatedness [Q model (green line), K model (red line) and Q + K model (yellow line)].** All 459 comparisons evaluated in 2011, 2012 and 2013 are considered. (TIFF 47 kb) Additional file 4:
**Correlation map for the traits evaluated in 2011, 2012 and 2013 seasons, based on the Pearson’s correlation coefficients.** The value of correlation (*r*) is shown according to color code. n.s.: not significant (*P* > 0.05). (TIFF 101 kb) Additional file 5:
***ΔK*** **plot for determining the number of genetic groups in the set of varieties considered in this study, obtained by means of STRUCTURE HARVESTER [** 82 **], based in the Evanno’s method [** 81 **].** (TIFF 72 kb) Additional file 6:
**Multiple regression analysis between phenotypic traits and population structure (genetic groups membership coefficients).** R^2^ indicates the proportion of explained variance. (XLSX 10 kb) Additional file 7:
**List of the 69 polymorphisms detected in the sequence of the** ***VvNAC26*** **gene in the set of grapevine varieties analized in this study.** (XLSX 14 kb) Additional file 8:
**Linkage disequilibrium (LD) among polymorphisms detected in the** ***VvNAC26*** **gene sequence. Only the 30 polymorphisms with a MAF > 5 % are considered.** Upper triangle shows the significance (*P*-value), whereas the lower triangle shows LD (R^2^). Values are coded according to the color bar at the right side. Polymorphisms in the LD-blocks A, B, C, D and E are indicated according to color code. (TIFF 194 kb) Additional file 9:
**Diversity values and neutrality tests for the grapevine** ***VvNAC26*** **gene.** The number of individuals (n), haplotypes (H), segregating sites (S), nucleotide diversity (π), Watterson’s estimate (θ), and Tajima’s *D* and Fu and Li’s *D** tests of neutral evolution are shown for the 114 grapevine varieties included in this study and for the three genetic groups. (XLSX 9 kb) Additional file 10:
**Hierarchical clustering of the 26** ***VvNAC26*** **haplotypes (H1 – H26) on the basis of 69 (A) and 3 selected (W-962, IND-649 and Y117) polymorphisms (B).** HGA and HGB indicate the two haplogroups detected. In B, MH1, MH2, MH3, MH4 and MH5 indicate the different minihaplotypes found. The observed distances are rescaled to fall into the range of 1 to 25. The ratio of the rescaled distances within the dendrogram is the same as the ratio of the original distances. (TIFF 117 kb) Additional file 11:
**mRNA secondary structures predicted by RNAsnp [** 68 **] for the first exon of the** ***VvNAC26*** **gene sequence.** The two variants (C and U) detected for the mutation Y117 are shown (A and B, respectively), and local regions comprising from nucleotide 102 to 151 are highlighted in green (C-variant) and red (U-variant). Note that Y117 does not produce any differentiation between both mRNAs. (TIFF 95 kb) Additional file 12:
**Expression levels for** ***VvNAC26*** **(VIT_01s0026g02710, in red) and** ***VvPI*** **(VIT_18s0001g01760, in blue) for cv.** Corvina in different tissues and developmental stages (if reported, the modified E-L stage [53] is given between brackets). Expression data was obtained from Fasoli et al. [106], where a detailed list of the samples used can be found. Every column shows mean value of three replicas, whereas vertical lines indicate standard deviation. (TIFF 246 kb)
