# Supplementary figures and images for: Polymorphisms and minihaplotypes in the VvNAC26 gene associate with berry size variation in grapevine
Source: BMC Plant Biol. 2015 Oct 23;15:253. doi: 10.1186/s12870-015-0622-2 (PMC4618959; doi:10.1186/s12870-015-0622-2)

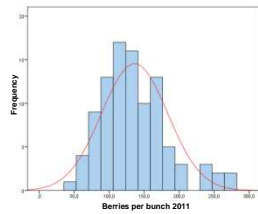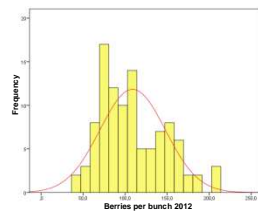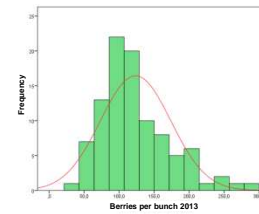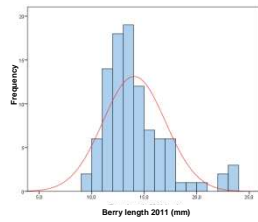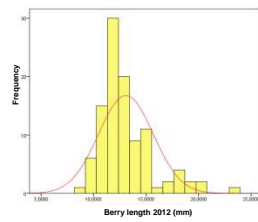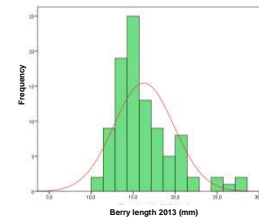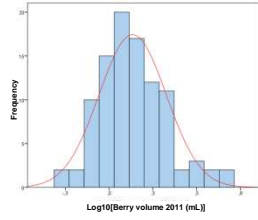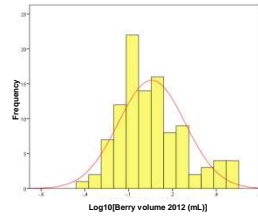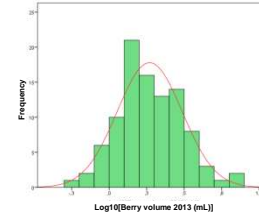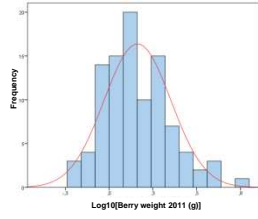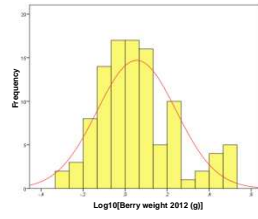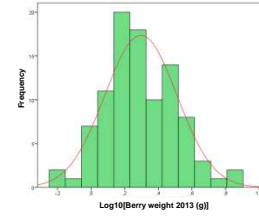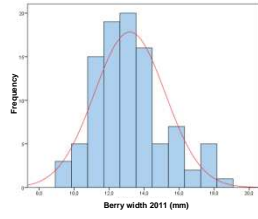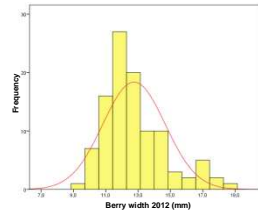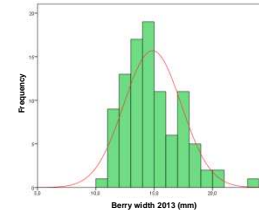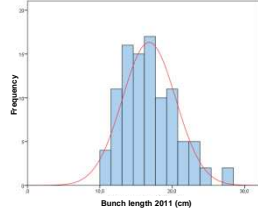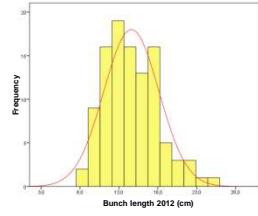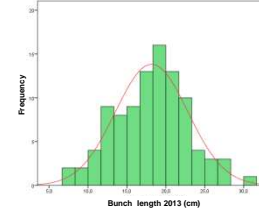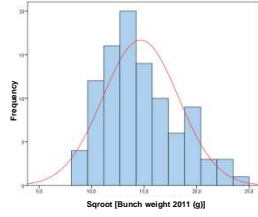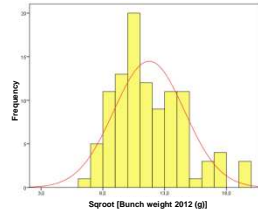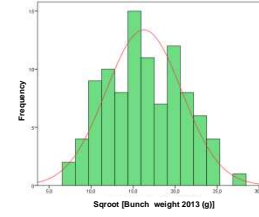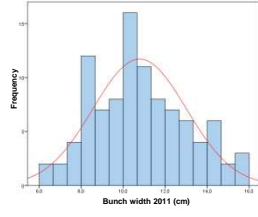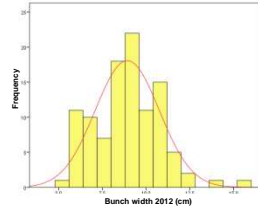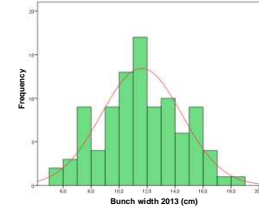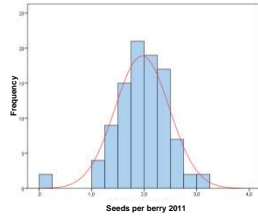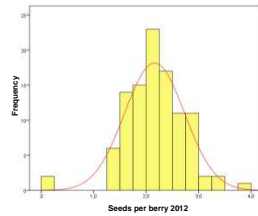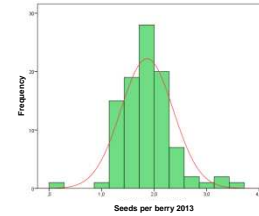

Supplement: Additional file 2: — Phenotypic distribution of the nine traits analyzed in this study for 2011 (skyblue), 2012 (yellow) and 2013 (green). (PDF 196 kb) [file 12870_2015_622_MOESM2_ESM.pdf]

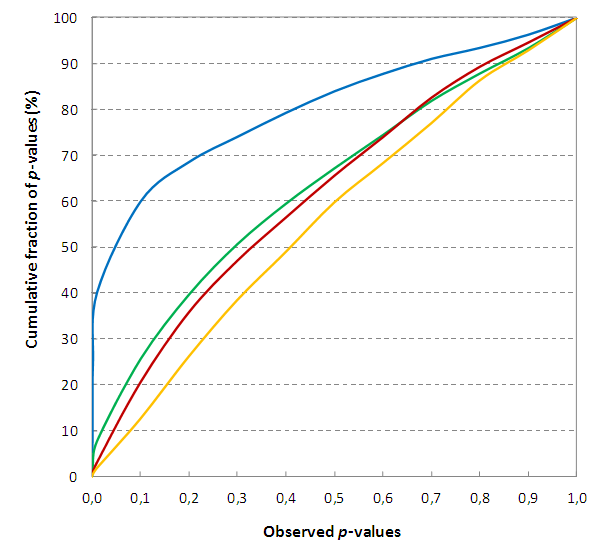

Supplement: Additional file 3: — Cumulative distribution of the P -values obtained for the trait-marker associations considering a naïve model (blue line) and three models controlling for different type of relatedness [Q model (green line), K model (red line) and Q + K model (yellow line)]. All 459 comparisons evaluated in 2011, 2012 and 2013 are considered. (TIFF 47 kb) [file 12870_2015_622_MOESM3_ESM.tif]

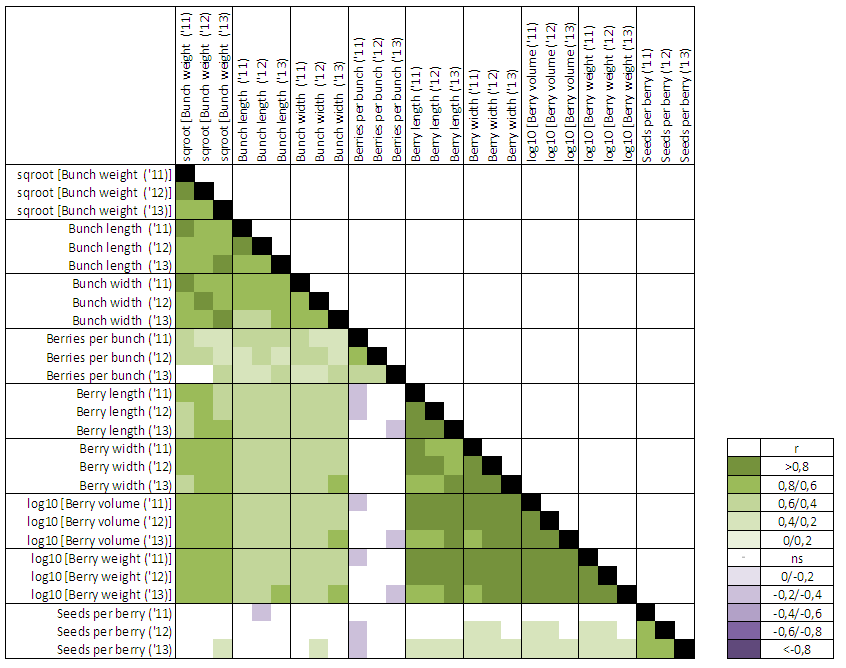

Supplement: Additional file 4: — Correlation map for the traits evaluated in 2011, 2012 and 2013 seasons, based on the Pearson’s correlation coefficients. The value of correlation (r) is shown according to color code. n.s.: not significant (P > 0.05). (TIFF 101 kb) [file 12870_2015_622_MOESM4_ESM.tif]

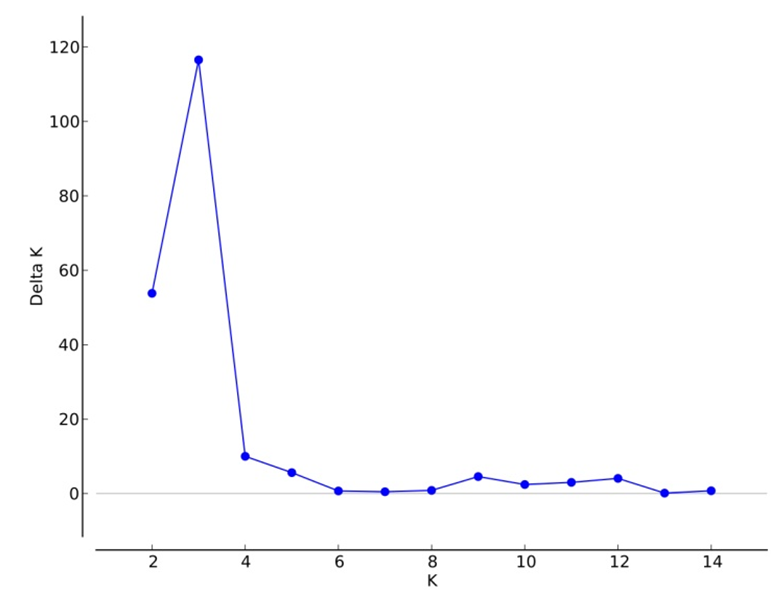

Supplement: Additional file 5: — ΔK plot for determining the number of genetic groups in the set of varieties considered in this study, obtained by means of STRUCTURE HARVESTER [ 82 ], based in the Evanno’s method [ 81 ]. (TIFF 72 kb) [file 12870_2015_622_MOESM5_ESM.tif]

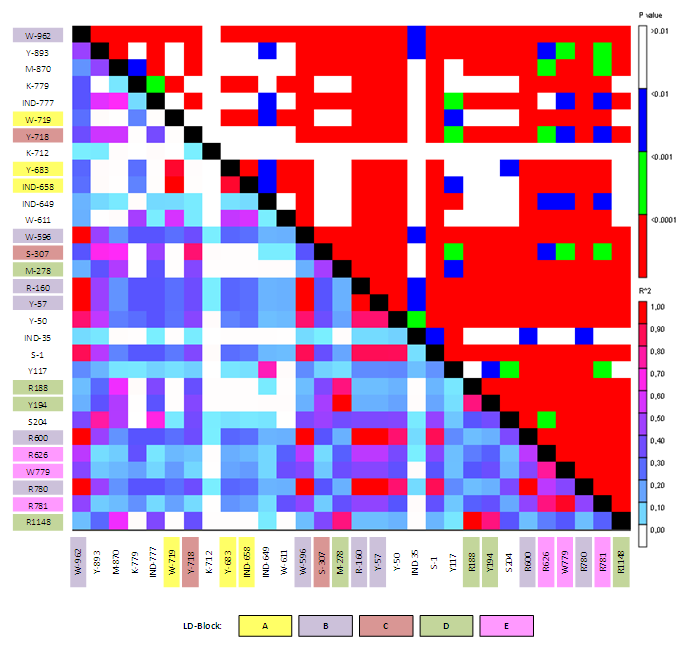

Supplement: Additional file 8: — Linkage disequilibrium (LD) among polymorphisms detected in the VvNAC26 gene sequence. Only the 30 polymorphisms with a MAF > 5 % are considered. Upper triangle shows the significance (P-value), whereas the lower triangle shows LD (R2). Values are coded according to the color bar at the right side. Polymorphisms in the LD-blocks A, B, C, D and E are indicated according to color code. (TIFF 194 kb) [file 12870_2015_622_MOESM8_ESM.tif]

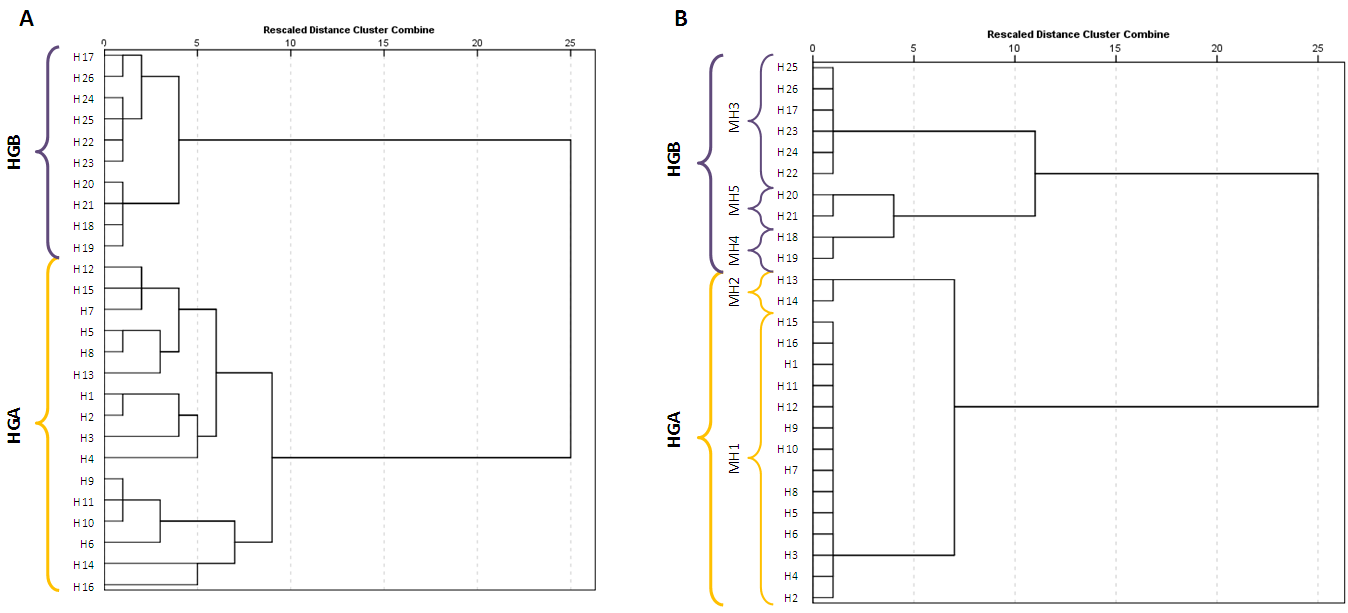

Supplement: Additional file 10: — Hierarchical clustering of the 26 VvNAC26 haplotypes (H1 – H26) on the basis of 69 (A) and 3 selected (W-962, IND-649 and Y117) polymorphisms (B). HGA and HGB indicate the two haplogroups detected. In B, MH1, MH2, MH3, MH4 and MH5 indicate the different minihaplotypes found. The observed distances are rescaled to fall into the range of 1 to 25. The ratio of the rescaled distances within the dendrogram is the same as the ratio of the original distances. (TIFF 117 kb) [file 12870_2015_622_MOESM10_ESM.tif]

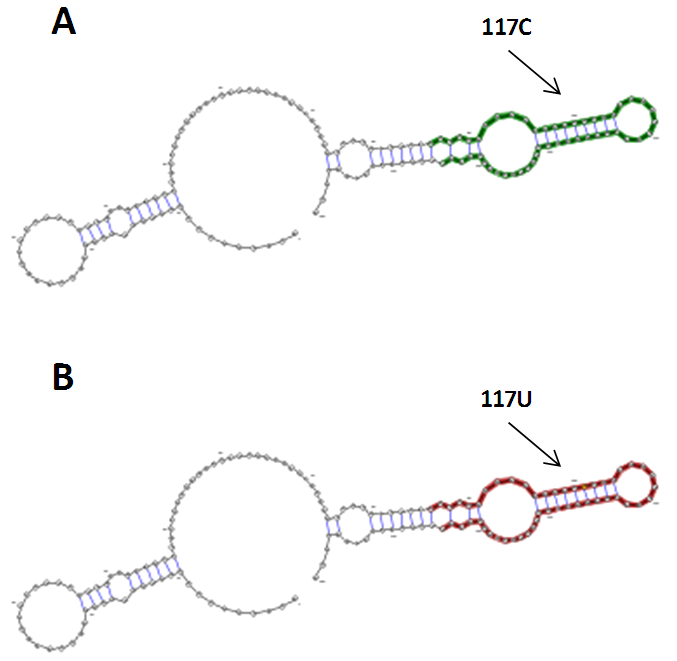

Supplement: Additional file 11: — mRNA secondary structures predicted by RNAsnp [ 68 ] for the first exon of the VvNAC26 gene sequence. The two variants (C and U) detected for the mutation Y117 are shown (A and B, respectively), and local regions comprising from nucleotide 102 to 151 are highlighted in green (C-variant) and red (U-variant). Note that Y117 does not produce any differentiation between both mRNAs. (TIFF 95 kb) [file 12870_2015_622_MOESM11_ESM.tif]

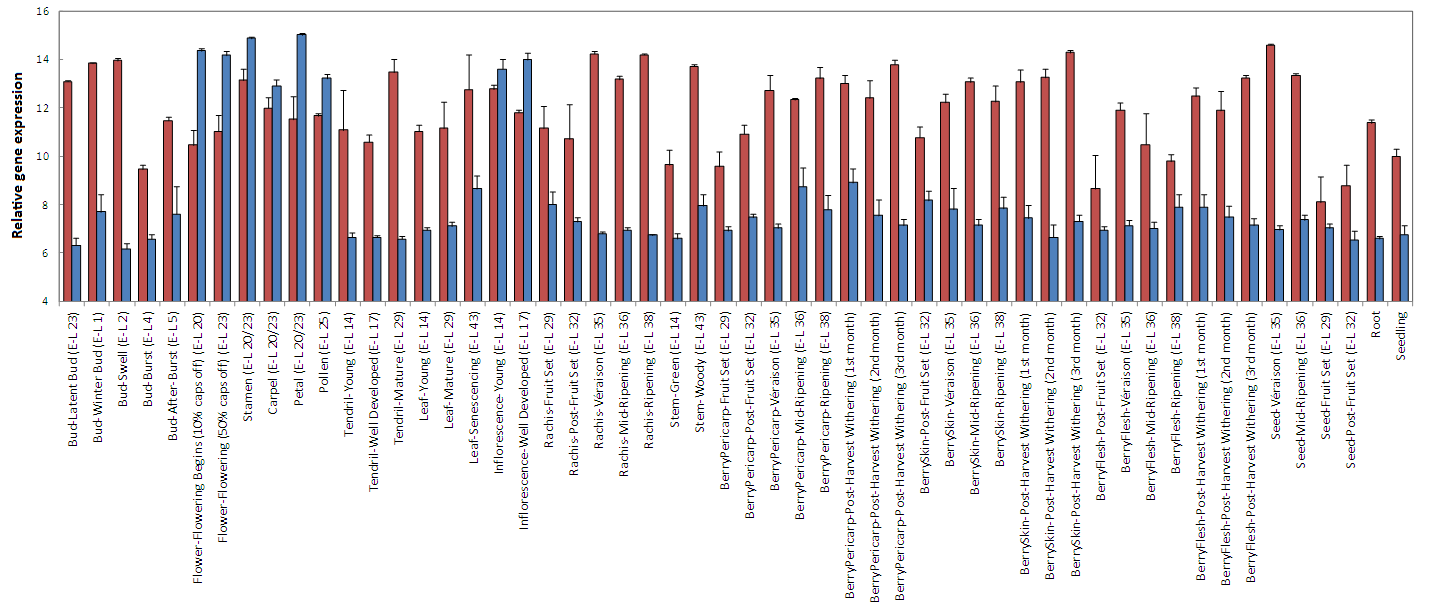

Supplement: Additional file 12: — Expression levels for VvNAC26 (VIT_01s0026g02710, in red) and VvPI (VIT_18s0001g01760, in blue) for cv. Corvina in different tissues and developmental stages (if reported, the modified E-L stage [53] is given between brackets). Expression data was obtained from Fasoli et al. [106], where a detailed list of the samples used can be found. Every column shows mean value of three replicas, whereas vertical lines indicate standard deviation. (TIFF 246 kb) [file 12870_2015_622_MOESM12_ESM.tif]
